# Protein re-surfacing of *E. coli* L-Asparaginase to evade pre-existing anti-drug antibodies and hypersensitivity responses

**DOI:** 10.3389/fimmu.2022.1016179

**Published:** 2022-12-07

**Authors:** Ali Bootwala, Hyun Hwan An, Meghan Whitney Franklin, Benjamin J. Manning, Lucy Y. Xu, Shruti Panchal, Joseph D. Garlick, Reshica Baral, Michael E. Hudson, Gevorg Grigoryan, Mark A. Murakami, Kristen Hopson, Daniel S. Leventhal

**Affiliations:** ^1^ Generate Biomedicines, Somerville, MA, United States; ^2^ Department of Medical Oncology, Dana-Farber Cancer Institute, Boston, MA, United States

**Keywords:** immunogenicity, anti-drug antibodies (ADA), hypersensitivity - immunology, protein engineering, computational protein design, asparaginase

## Abstract

The optimal use of many biotherapeutics is restricted by Anti-drug antibodies (ADAs) and hypersensitivity responses which can affect potency and ability to administer a treatment. Here we demonstrate that Re-surfacing can be utilized as a generalizable approach to engineer proteins with extensive surface residue modifications in order to avoid binding by pre-existing ADAs. This technique was applied to *E. coli* Asparaginase (ASN) to produce functional mutants with up to 58 substitutions resulting in direct modification of 35% of surface residues. Re-surfaced ASNs exhibited significantly reduced binding to murine, rabbit and human polyclonal ADAs, with a negative correlation observed between binding and mutational distance from the native protein. Reductions in ADA binding correlated with diminished hypersensitivity responses in an *in vivo* mouse model. By using computational design approaches to traverse extended distances in mutational space while maintaining function, protein Re-surfacing may provide a means to generate novel or second line therapies for life-saving drugs with limited therapeutic alternatives.

## Introduction

Unwanted immunogenicity, particularly the presence of anti-drug antibodies (ADAs), remains a critical challenge to the clinical application of proteins to treat human disease. Whether pre-existing or treatment emergent, ADAs can adversely impact therapeutic effect through direct neutralization of protein function or accelerated drug clearance thereby reducing effective exposure time. In worst-case scenarios, induction of hypersensitivity and anaphylaxis responses can ultimately prevent a patient from being eligible to receive lifesaving treatments.

Pre-existing ADAs can result from prior exposure to microbes, pathogens and biotherapeutics. For example, the high prevalence of neutralizing antibodies against the common gene delivery vector adeno-associated virus (AAV) is thought to be the result of lifelong exposure to natural AAVs (1). Emergence of ADAs against therapeutically useful toxins can occur following routine vaccination, like the development of anti-Diphtheria toxin antibodies following vaccination with the Tetanus-Diphtheria-Pertussis (Tdap) vaccine. These ADAs pose a particular challenge for therapeutic immunotoxins, such as Denileukin diftitox (Ontak^®^) and Tagraxofusp (Elzonris^®^), which utilize Diphtheria toxin as an oncolytic agent (2, 3). While the above examples highlight potential causes of ADAs, most ADAs against biotherapeutics emerge post treatment with the therapeutic molecule itself. In all cases, a generalizable approach to abrogate drug binding to and evasion of downstream functional consequences of ADAs would confer significant clinical value by providing a means to generate second line therapeutic options.

Multiple strategies have been proposed to mitigate hypersensitivity reactions associated with ADAs, including concomitant immunosuppression and hyper dosing to induce drug desensitization (4). Neutralizing ADAs can also be targeted by IgG-cleaving endopeptidases, like Imlifidase which previously had been utilized to induce organ tolerance in transplant patients (5). A more direct approach would be to eliminate the ADA epitopes on the surface of the biotherapeutic and thereby impair binding by pre-existing antibodies. Traditional antibody epitope mapping approaches utilize polyclonal ADAs binding to whole protein and methods such as x-ray crystallography, hydrogen-deuterium exchange, competitive binding assays or mutational scanning (6). Unfortunately, these approaches are extremely costly, low throughput, time intensive and can fail to identify all potentially relevant epitopes. Conversely, while the use of computational antibody epitope predictors would be faster and higher throughput, current models primarily focus on linear epitopes and have low accuracy with high false positive rates (7).

Even if all potential B-cell epitopes were accurately identified, the sheer number of modifications required to disrupt all epitopes without disrupting protein folding and function remains a significant engineering challenge. B-cell epitope removal *via* widespread surface residue modification has been previously applied towards the objective of immunofocusing in vaccine design. Glycan masking is one approach that involves the insertion of glycosylation sites to disrupt nearby antibody epitopes (8, 9). This approach could be limited for therapeutic proteins due to disruption of function, due to unwanted gain of functions, or for proteins produced in systems lacking glycosylation machinery (such as bacteria). Direct modification of protein sequence *via* domain deletion and surface residue mutation is another approach employed in vaccine design. After grafting the 4E10 HIV epitope onto an *E. coli* derived scaffold protein, Correia et al. removed potentially immunodominant epitopes from the scaffold using domain trimming and protein resurfacing to make a soluble, stabilized antigen capable of focusing the immune response to the epitope of interest (10). While these studies demonstrate the use of surface residue modification for focusing an immune response to specific, desired antibody epitopes, we hypothesized that surface modification can also be applied as an approach to avoid pre-existing ADA binding against therapeutic proteins. We therefore set out to utilize computational protein design to broadly modify surface residues while preserving protein folding and function. Here we utilize the oncolytic enzyme L-Asparaginase (ASN) as a clinically relevant model to highlight the utility of Re-surfacing for avoiding binding by pre-existing ADAs.

L-Asparaginase is a standard component of many treatment regimens for pediatric and adult acute lymphocytic leukemia (ALL) (11, 12). First line clinical ASN preparations are derived from *Escherichia coli* (*E. coli*) and administered in either a native (Elspar^®^) or polyethylene glycosylated (PEG) (Oncaspar^®^ and Asparlas^®^) format. Exposure to either version of ASN can induce a myriad of adverse events such as, hepatitis, pancreatitis, thrombosis, and hypersensitivity reactions, including anaphylaxis (13). Approximately 22-30% of patients receiving *E. coli* ASN based treatments exhibit hypersensitivity responses and 8-18% develop silent inactivation with the emergence of neutralizing ADAs without overt hypersensitivity (14–17). Hypersensitivity and treatment emergent ADAs can ultimately limit patients’ overall dose exposure and have been associated with inferior event-free survival (18). Patients in the United States who develop allergic reactions to *E. coli* ASN are frequently switched to *Erwinia Chrysanthemi* derived ASN (Erwinaze^®^), but up to 33% of such patients will go on to develop hypersensitivity to *Erwinia* ASN treatment as well (19). Furthermore, while *Erwinia* ASN treatment may be effective in permitting ongoing ASN dosing, its efficacy is still limited by a shorter half-life, lower remission rates, and manufacturing constraints (20–22). L-Asparaginase therefore constitutes a therapeutic exemplar for which Re-surfacing could facilitate generation of novel mutants (hereby referred to as variants) that overcome the effects of pre-existing ADAs that emerged following treatment using the native protein.

Here we describe a generalizable approach to design therapeutic proteins with reduced binding to pre-existing ADAs. We utilize previously described structure-guided, machine learning-based methods (23, 24) to disrupt ADA binding by replacing the maximum number of surface amino acid residues, thereby modifying potential ADA epitopes, while maintaining protein expression and function. Because protein Re-surfacing has many potential applications in oncology, we selected ASN as a prototypical non-human protein therapeutic for which dose exposure is critical for optimal clinical response but limited at times by immunogenicity. Re-surfaced ASNs (Res. ASNs) were evaluated for relative expression and activity compared to the wild type *E. coli* ASN (WT ASN). The relative binding of Res. ASNs against murine and rabbit polyclonal ADAs were then assessed and compared to WT ASN. To evaluate the impact of Re-surfacing on binding to human ADAs, serum and plasma samples from 10 ALL patients who previously exhibited hypersensitivity responses were determined. Relative binding of murine, rabbit and human Res. ASNs were compared to the mutational distance of each variant from WT ASN to begin establishing general principles for the number of mutations required to drive down binding by polyclonal ADAs. Finally, Res. ASNs were evaluated for their ability to alleviate hypersensitivity responses in mice previously sensitized to WT ASN to determine if the observed reductions in ADA binding were sufficient to make a meaningful biological impact *in vivo*. Together these results demonstrate the capability of computational directed engineering to identify suitable amino acid substitutions and enable the generation of functional ASN variants with significantly reduced binding to ADAs and ameliorated hypersensitivity responses. Protein Re-surfacing represents a generalizable approach to avoid pre-existing ADA binding and may facilitate the development of novel and second line therapies for critical treatments for which additional options are urgently needed.

## Results

### Computational generation of re-surfaced ASNs

**Figure 1:**
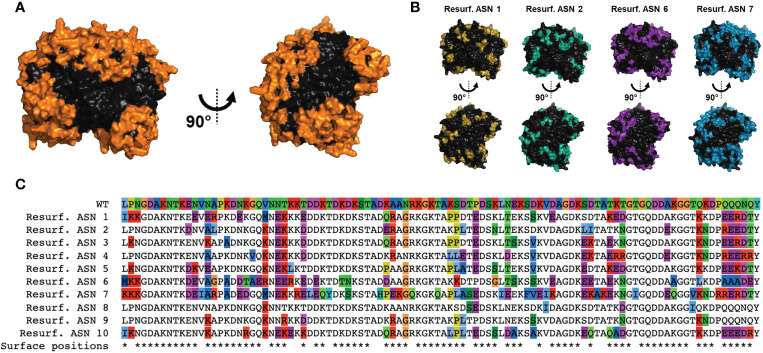
Computational design of Re-surfaced ASN variants. Variant proteins were designed referencing wild-type *E coli* asparaginase (WT ASN) (PDB ID: 3ECA) with mutations focused around residues with a relative solvent accessibility >50% (referred to as surface exposed residues). **(A)** Structural view of the WT ASN homotetramer with amino acid positions open to mutation shown in orange. **(B)** Four representative Resurf. variants with colored positions indicating residues with mutations differing from WT ASN. **(C)** Multiple sequence alignment of the Resurf. ASN designs showing surface exposed and mutated positions. The top row shows the WT ASN sequence with all positions colored by amino acid characteristic (polar in green, nonpolar in blue, positively charged in red, negatively charged in purple, aromatic in cyan, and proline, cysteine, and glycine in unique colors). The bottom row indicates positions classified as solvent exposed and are denoted with an asterisk.

To generate Res. *E. coli* ASN variants, structure-based computational approaches were utilized with the design goal of maximal differentiation of surface-exposed residues while maintaining enzymatic function. To accomplish this, crystal structures of *E. coli* ASN (PDB ID 3eca) were utilized to create a structure-based predictor as a proxy for function. To maximize the probability of disrupting ADA epitopes, mutations were focused on or in direct proximity to amino acids with >50% solvent accessibility. In total, 78 of the 326 residues were considered surface exposed. To avoid disruption of protein fold and enzymatic function, mutations were also restricted to positions that were not within or in direct proximity to the enzymatic core or monomer-monomer interfaces. Based on these specifications 85 residues were available for modification (**Figure 1A** and **Supplementary Figures S1A, B**), 46 of which were considered as surface exposed. Candidate sequences were selected from a larger pool of sequence proposals based on functional prediction scores and to maximize sequence diversity across all variants nominated for testing. Candidate variants exhibited a wide range of mutations in relation to residue position and amino acid substitutions made, with 68 of the 85 designable residues mutated in at least one of the candidates. This is exemplified and illustrated for 4 of the top candidates *via* structural rendering (**Figure 1B**) and for all 10 candidates *via* a multiple sequence alignment (**Figure 1C**). Variant sequences contained between 6 to 58 substitutions, resulting in 96-82% total similarity or 94-65% surface-residue similarity to wild type (WT) *E. coli* ASN, hereby referred to as WT ASN (**Table 1**).

**Table 1:** Mutational distance and percent homology for Re-surfaced ASN candidates.

Re-surfaced ASN Number	Number of Mutations (Total)	Percentage of AA’s Mutated (Total)	Percentage of AA’s Mutated(Surface Exposed)
**1**	**29**	**9%**	**14%**
**2**	**26**	**8%**	**14%**
**3**	**27**	**8%**	**14%**
**4**	**26**	**8%**	**13%**
**5**	**27**	**8%**	**14%**
**6**	**39**	**12%**	**19%**
**7**	**58**	**18%**	**35%**
**8**	**6**	**2%**	**6%**
**9**	**12**	**4%**	**6%**
**10**	**31**	**10%**	**14%**

Values shown refer to mutations within one of the four monomers which form the WT ASN homotetramer. The total number, percent of all amino acids (AAs) and percent of surface exposed AAs (with solvent accessibility >50%) differing from the WT ASN sequence are shown. WT ASN has 78 residues considered to be surface exposed with some mutations falling outside of these residues.

### Re-surfaced ASNs maintain enzymatic function

**Figure 2:**
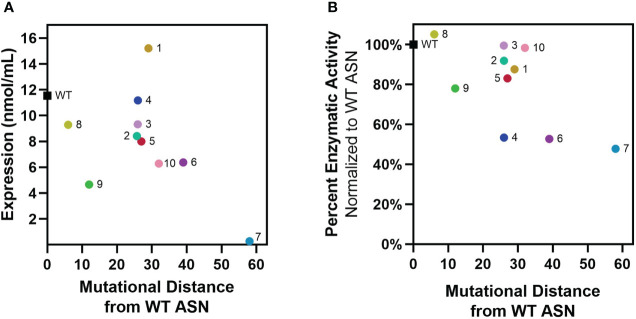
Re-surfaced ASNs express and maintain activity at high mutational loads. Re-surfaced ASN variants were expressed in *E coli*, with protein abundance and enzymatic activity quantified from bacterial lysates. **(A)** Recombinant ASN protein was measured *via* His-tag quantification, with expression compared to mutational distance from WT ASN. **(B)** Recombinant ASN enzymatic activity was measured *via* biochemical kinetic assay following binding to nickel-coated plates. Relative activity compared to WT ASN is shown and compared to mutational distance from WT ASN. All data points represent the mean values from 3-4 technical replicates.

Re-surfaced and control WT ASN sequences were cloned to contain a N-terminal multi-histidine tag to enable measurement of expression and activity directly from bacterial supernatants while differentiating from the low levels of WT ASN endogenously expressed by the *E. coli* host strain. Additionally, multi-histidine tagging was later utilized to facilitate enrichment and purification in large scale cultures. Placement of the multi-histidine tag on the N or C-terminus was first evaluated, with fusion on the C-terminus resulting in significant reductions in expression and/or activity while a N-terminal tag placed directly after the cleaved periplasm signal sequence was found to be permissive. Following recombinant protein induction, *E. coli* ASN expression and activity were measured. While variants containing the largest number of mutations typically exhibited the lowest overall expression, Res. ASN 1 showed higher levels of expression than WT (**Figure 2A**). To differentiate enzymatic activity of the recombinantly expressed ASNs versus the endogenous protein, bacterial lysates were bound to anti-His tag coated plates, rinsed, and assessed for ASN activity (**Figure 2B**). Despite containing large numbers of mutations, Res. ASN’s exhibited at least 50% enzymatic activity compared to recombinant WT ASN, suggesting proper folding and formation of active homotetramers.

These data demonstrate that computational approaches to protein re-surfacing can generate expressible and functional protein variants with high sequence diversity and large numbers of surface mutations (up to 58 amino acids away from WT). Following confirmation of enzymatic activity, Res. ASN variants were moved on to evaluate relative binding to anti-drug antibodies from mice, rabbits, and humans.

### Reduction in binding to mouse and rabbit anti-ASN antibodies

To evaluate the ability of Res. ASNs to avoid binding to pre-existing ADAs, relative binding of polyclonal mouse and rabbit antibodies from immunized animals were tested. Plasma containing high titers of polyclonal mouse anti-ASN antibodies were produced by immunizing Balbc mice against WT ASN complexed to alum adjuvant. Polyclonal rabbit anti-ASN was available commercially. While the epitope specificities of the polyclonal anti-ASN antibodies were unknown, we hypothesized that widespread modification of surface residues with up to 58 mutations from WT would be sufficient to impact overall antibody binding.

**Figure 3:**
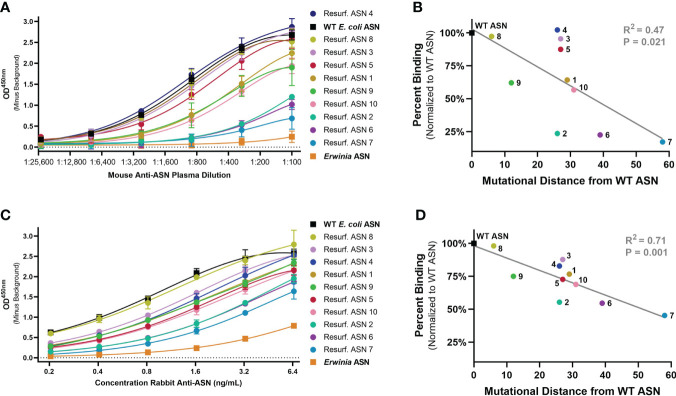
Re-surfaced ASN’s exhibit reduced binding to polyclonal mouse and rabbit ADAs. An enzyme-linked immunosorbent assay (ELISA) was utilized to monitor relative binding of polyclonal murine and rabbit ADAs to Resurf. ASN variants. Relative binding curves as shown with raw OD^450^ values across serial dilutions of **(A)** primary mouse plasma or **(C)** purified rabbit antibodies with mean and standard deviation shown for technical triplicate measures. ASNs are listed in order of highest to lowest titer with WT ASN shown as a black box, the negative benchmark control *Erwinia* ASN shown as an orange box. The mean binding normalized to the WT ASN control of **(B)** mouse serum at a 1:300 dilution and **(D)** polyclonal rabbit anti-ASN ADAs at 3.2 ng/mL to each ASN variant is plotted against the mutational distance from WT ASN. Linear regression analysis demonstrates a significant negative correlation between binding and mutational distance across both species, R^2^ = 0.47 and *P*<0.05 for mouse serum and R^2^ = 0.71 and *P* = 0.001 for rabbit ADAs. Data are representative of N = 2 independent experiments.

Binding of murine polyclonal ADAs was measured using an enzyme-linked immunosorbent assay (ELISA) with an anti-murine IgG secondary. Wildtype ASN exhibited the highest levels of binding while the benchmark control *Erwinia* derived ASN (having approximately 48% homology to *E. coli* ASN, the equivalent of 176 mutations) had the lowest levels of binding (**Figure 3A**). Re-surfaced ASN variants exhibited a range of binding which negatively correlated with the number of mutations from WT ASN (**Figure 3B**). Binding against rabbit polyclonal ADAs were measured in a similar manner utilizing an anti-rabbit IgG secondary. Polyclonal rabbit ADAs binding to WT ASN, *Erwinia* ASN control and the Res. ASN variants showed similar ranking to murine ADAs, with relative binding negatively correlated with the number of mutations away from WT ASN (**Figures 3C, D**).

### Decreased binding by human anti-ASN antibodies from ALL patients

With similar reductions in ADA binding observed for polyclonal murine and rabbit antibodies, we next assessed binding to human ADAs from adult ALL patients. To identify clinical samples with the greatest likelihood of containing high titers of anti-ASN ADAs, we reviewed the clinical records associated with appropriately consented ALL patient samples within a biospecimen repository at the Dana-Farber Cancer Institute (DFCI). From a query of 385 adult ALL patients with at least one banked specimen, clinical abstraction revealed 50.6% (195) to have received an asparaginase treatment, of which 35.3% (69) exhibited some level of adverse reaction with 20% (25) experiencing acute onset of illness involving skin and mucosal tissue or respiratory compromise. From the list of 39 patients, 26 had samples banked between the dates of allergic reaction and allogenic transplant, a time point when ADA titers would most likely be present. Twenty of these samples were tested for the presence of anti-ASN IgG antibodies against WT ASN using an ELISA-based binding assay.

**Figure 4:**
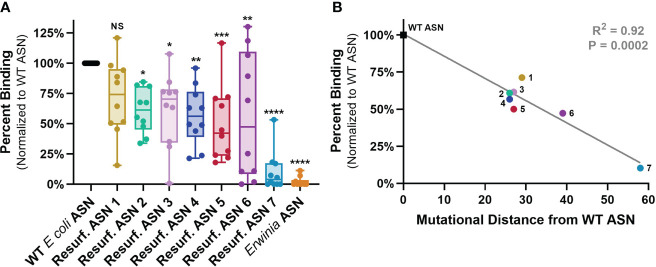
ASN Re-surfacing disrupts binding to human ADAs. Anti-ASN ADA titers were quantified against WT ASN for each sample *via* ELISA with the dilution exhibiting 80% of max binding signal utilized to measure relative binding to Resurf. ASN variants (See **Supplementary Figure 1**). **(A)** Human serum or plasma sample binding to Resurf. ASNs normalized to binding against WT ASN as assayed by IgG ELISA. Each dot is the mean of an individual patient sample tested in technical triplicate. All ASN variants except Resurf. ASN 1 exhibited significant reductions in normalized binding *via* one-way analysis of variance (ANOVA), *P < 0.05, **P < 0.01, ***P < 0.001, ****P < 0.0001. **(B)** The mean normalized percent binding across all patient samples to each ASN is plotted against the mutational distance of the molecule to WT ASN and shows a significant negative correlation *via* linear regression analysis, R^2^ = 0.92, *P* = 0.0002. Data are representative of N = 3 independent experiments. Please see **Supplementary Figure 2** for binding assessment normalized to relative abundance of plate bound protein measured by anti-His ELISA. NS, not significant p>0.05.

Of the twenty samples tested, a total of five plasma and six serum samples exhibited high titers of ADAs against WT ASN (with positive signal above background at up to a 1:1,000,000 dilution) (**Supplementary Figure S2**). These samples were nominated for testing against Res. ASNs (patient summary data shown in **Table 2** and further detailed information found in **Supplementary Table S1**). Patient serum or plasma were titrated to identify the concentration which exhibited approximately 80% of max binding signal for each sample, while exhibiting minimal background signal from pooled healthy donor serum or plasma at similar concentrations (**Supplementary Figure S2**). The identified dilution was then used to assess relative binding against Res. ASNs compared to WT. In agreement with binding patterns observed from murine and rabbit ADAs, Res. ASNs exhibited an overall reduction in binding to human ADAs compared to WT ASN (**Figure 4A**) and binding was negatively correlated with the number of mutations from WT ASN (**Figure 4B**). To ensure reductions in ADA binding signal were not due to reduced binding of Res. ASN proteins to the ELISA plates, we utilized the multi-histidine tags shared by all proteins to quantify relative abundance of protein *via* an anti-His tag ELISA. When ADA binding signal was normalized to the anti-His quantification signal, similar or further reductions in ADA binding were observed (**Supplementary Figures S3A, B**). Other than one patient sample which was excluded following outlier analysis (see Materials and Methods), no single sample consistently displayed the highest or lowest level of binding across all ASN’s (**Supplementary Table S2**). These data imply that many epitopes are important for overall binding by human ADAs and highlight the diverse polyclonal response represented amongst the patient samples analyzed.

**Table 2:** Summary of patient descriptors for samples with detectable anti-asparaginase IgG antibodies.

	Plasma (n = 5)	Serum (n = 6)	Total (n = 11)
**Age (years)**	47.6 ± 4.5	42.0 ± 6.0	44.5 ± 12.5
**Sex**			
Male	3	5	8
Female	2	1	3
**Diagnosis**			
B-ALL	4	5	9
T-ALL	1	1	2
Therapy Regimen*			
CALGB-9111 (Larson)	4	5	9
POG-9900^‡^	0	1	1
DFCI 15-709	1	0	1
**Lines of Therapy**			
First	4	5	9
Second	1	1	2
**ASN Formulation**			
*E. coli*	1	5	6
PEG	4	1	5
**Days of ASN Treatment until reaction**	40.0 ± 18.1 (14 – 107)	24.0 ± 7.6 (0 – 41)	31.0 ± 9.1
**Number of days post-reaction**	55.2 ± 16.0 (11 – 106)	349.8 ± 142.5 (18 – 939)	215.9 ± 87.9
**Rheumatological Diagnoses**	0	0	0
**History of drug-induced allergic reactions**	2	5	7

Patient characteristics were abstracted from electronic clinical records and shown here for all patients with detectable anti-ASN ADAs. Please see **Supplemental Table 1** for more detailed clinical information.

These data provide support for the utility of protein Re-surfacing to disrupt binding by highly diverse, polyclonal pre-existing ADAs from mouse, rabbit and humans. The observed correlations between mutational distance and ADA binding also imply that further mutations could drive binding even lower.

### Re-surfaced ASNs ameliorate treatment related hypersensitivity

**Figure 5:**
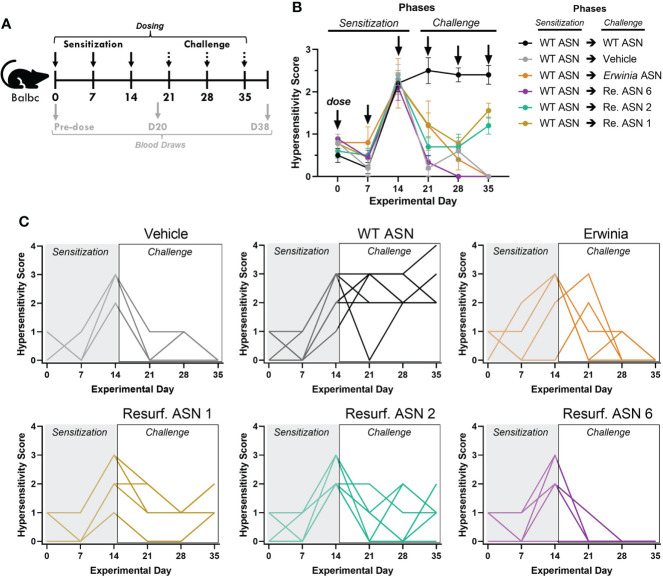
Resurf. ASNs alleviate treatment related hypersensitivity in mice. **(A)** Schematic of sensitization and hypersensitivity challenge model. Wildtype Balbc mice were given three intravenous (IV) sensitization doses of WT ASN (50µg per dose) after which mice were randomized across experimental groups to normalize hypersensitivity scores. Mice were then given three IV challenge doses of Vehicle control, WT ASN as a positive control, *Erwinia* ASN as a negative benchmark control, or one of three selected Resurf. ASN variants. Hypersensitivity reactions were monitored for the first 30 minutes post-dose and quantified on a four-point scale based on severity (see Materials and Methods for further details). Blood was drawn and serum harvested on Days 0 (pre-dose), 20 (prior to initiation of challenge phase) and 38 (at the completion of the study) to quantify emergence and titer of ADAs (see **Supplementary Figure 3**). **(B)** The mean and standard error mean for hypersensitivity scores following each dose for each experimental group are shown. See **Table 3** for statistical comparison of each experimental group compared to WT ASN for each dose of the challenge phase. **(C)** The hypersensitivity scores recorded for each individual mouse is shown for each respective experimental group. Data are shown for an N = 1 experiment.

The emergence of acute hypersensitivity or anaphylactic responses is one clinically debilitating consequence of ADA formation which most often requires discontinuation of a treatment. Balbc mice recapitulate a variety of features of the hypersensitivity observed during treatment with *E. coli* ASN in humans (26). We thus sought to evaluate if the observed reductions in ADA binding to Res. ASNs were sufficient to alleviate hypersensitivity following sensitization to WT ASN in mice. To induce sensitization, WT ASN was intravenously administered once a week for three weeks to Balbc mice (**Figure 5A**). On the fourth week cohorts of mice were then challenged with either WT ASN (as positive control), vehicle alone (as negative control), *Erwinia* ASN (as a benchmark control) or Res. ASNs. Clinical observations of acute hypersensitivity reactions were noted and quantified using a 5-point severity scale (27). Hypersensitivity reactions were 100% penetrant at the completion of the sensitization phase following the third dose (**Figures 5B, C**). Mice receiving doses of WT ASN during the challenge phase continued to exhibit high levels of hypersensitivity. Cohorts transitioned to challenge with Res. ASNs exhibited a significant reduction in hypersensitivity responses, comparable to the vehicle only and *Erwinia* ASN controls (**Table 3**). Comparable to the *Erwinia* ASN benchmark, Res. ASN 6, having the most mutations away from WT ASN of the three Res. ASNs tested (**Table 1**), showed the largest reductions in hypersensitivity with no mice displaying reactions following the final third dose of the challenge phase (**Figure 3C**). Similar patterns were observed for the emergence of anti-ASN ADAs at the conclusion of the study, with low or no detected titers against *Erwinia*, Res. ASN 2 and Res. ASN 6, moderate titers against Res. ASN 1 and high titers against WT ASN (**Supplementary Figure S4A**). Finally, challenge with Res. ASNs or *Erwinia* ASN did not result in decreases of ADAs targeted to WT ASN (**Supplementary Figure S4B**).

**Table 3:** Resurf. ASNs significantly reduced hypersensitivity responses *In vivo*.

Sensitization Group	Challenge Group	Number of mice	*P*-value from one-way ANOVA compared to WT ASN challenge group at indicated timepoint		
			*Day 21*	*Day 28*	*Day 35*
WT ASN	**Vehicle**	N = 5	<0.0001	<0.0001	<0.0001
WT ASN	***Erwinia***	N = 5	0.0248	<0.0001	<0.0001
WT ASN	**Resurf. ASN 6**	N = 9	<0.0001	<0.0001	<0.0001
WT ASN	**Resurf. ASN 2**	N = 10	<0.0001	<0.0001	<0.0001
WT ASN	**Resurf. ASN 1**	N = 9	0.0065	<0.0001	0.0027

Summary of experimental replicates and p-values from one-way ANOVA comparing hypersensitivity scores of experimental groups against mice receiving WT ASN during the challenge phase on the indicated days. All experimental groups exhibited significant reductions in hypersensitivity severity scores with a P <0.05.

Taken together, our results demonstrate the ability of protein Re-surfacing to reduce binding to pre-existing ADAs and to a degree sufficient to blunt *in vivo* hypersensitivity responses.

## Discussion

In this study we demonstrate the ability of protein Re-surfacing, enabled by computational design using structure-informed algorithms, to generate proteins that maintain function while containing high numbers of mutations away from the native sequence. We show that the widespread modification of solvent-exposed residues, without the need to identify the epitopes targeted by ADAs *a priori*, reduces binding by pre-existing ADAs from three independent species (including human). We further demonstrate that Res. ASN variants can mitigate hypersensitivity reactions observed in mice previously sensitized to the WT protein, suggesting that the reductions in ADA binding observed *in vitro* are sufficient to make a meaningful biological impact *in vivo*.

Here we show the ability of our protein Re-surfacing approach to identify mutations which disrupt ADA binding epitopes while maintaining protein function, even for a complex homotetrameric protein like *E. coli* ASN. While the Res. ASN variants reported here did not take any previously identified antibody epitopes into account during their design, several epitopes reported in the literature were targeted *via* our broad approach (**Supplementary Figure S5**). Seven of the eleven reported human B cell epitopes previously identified *via* measurement of binding to human polyclonal anti-ASN antibodies contained one to several mutations away from WT ASN (28, 29). Additionally, two of the three locations directly engineered to reduce binding by mouse and rabbit ADAs by other groups were also targeted by several of our Res. ASN variants (29, 30).

Our *ex vivo* data utilizing mouse, rabbit and patient samples demonstrate a reduction in anti-ASN antibody binding for IgG isotypes. While previous studies have demonstrated that IgG can mediate hypersensitivity reactions, IgE isotype ADAs are thought to be a primary driver of anaphylaxis *via* the activation of mast cells (31). The short half-life of IgE antibodies often leads to rapid clearance and an inability to detect ADAs of this isotype in primary specimens from mouse models and human patients. Additionally, in the absence of a biological rationale to support the idea that IgG and IgE antibodies bind to distinct antigenic epitopes, measurement of IgG binding should be relatively representative of other high affinity isotypes. These conclusions are further supported by the *in vivo* validation studies reported here demonstrating that Res. ASN variants alleviate hypersensitivity responses in mice sensitized to WT ASN. If IgE antibodies were driving hypersensitivity in this mouse model, Res. ASNs would thus need to avoid binding by IgEs to alleviate said phenotype.

While previous studies demonstrated the feasibility of targeting individual identified epitopes, the primary practical challenge is that widespread disruption of all potential epitopes requires a substantial number of mutations. Antibody responses to SARS-COV-2 represent a contemporary example of such high mutational loads being required to substantially reduce the impact of polyclonal antibody responses. Polyclonal sera from convalescent patients infected with or receiving vaccination with spike from the wild-type parental SARS-COV-2 strain demonstrated significant loss in neutralization activity to variants of the Omicron strain, with said variants containing 29-34 mutations within their spike domains (32). As demonstrated here, anywhere from 26-58 mutations are required to reduce ADA binding to below 50% relative to the WT protein. Previous attempts to create engineered variants of *E. coli* ASN using traditional approaches such as single point mutations and/or directed evolution were only able to achieve 2-9 mutations away from the native protein (30, 33, 34). Other groups employing computational design methods to resurface proteins have achieved significantly high levels of modification with up to 49 mutations or 42% of a 116 amino acid protein consisting of a three-helix bundle structure (10). Here we show soluble, functional enzyme variants with up to 58 mutations from the native 326 amino acid sequence for a highly complex homotetrameric protein, a new milestone for protein engineering. Our data also suggest that further reductions in ADA binding may be possible by mutating additional surface residues to further reduce similarity to the native protein. The marginal reductions in enzymatic activity and expression observed with increasing mutational burden suggests a potential benefit to subsequent quantitative modeling of the impact of individual surface residues to facilitate further optimization between the trade-offs of immunogenicity, function, and expression with higher resolution. However, in this study the lack of single point mutation mutants and relatively low number of variants evaluated would limit our ability to identify patterns that could enable further protein optimization. Future approaches utilizing this data, taking previously identified epitopes into consideration, co-optimizing for both function and expression, as well as incorporating machine learning techniques and large-scale design-test-learn cycles, could enable further improvements.

The results presented here provide strong support for further development, optimization, and use of Re-surfacing approaches to alleviate the deleterious, and at times pathogenic, effects of pre-existing immunity against lifesaving therapeutics. Re-surfacing may be particularly useful for enzyme therapeutics that are limited by a paucity of therapeutic alternatives and are constantly challenged by the balance between activity, specificity, developability and immunogenicity (35, 36). In addition to ASN, Re-surfaced coagulation factors could also provide benefit to hemophiliac patients as the efficacy of these lifesaving biotherapeutics are often limited over time by immune-mediated clearance and inactivation (37, 38). Conjugation of biocompatible chemical polymers, such as polyethylene glycol (PEG), has been utilized to both extend half-life and mask antibody epitopes on the surface of therapeutic proteins. One limitation of such approaches includes the high variability of chemical conjugation that results in a poorly controlled heterogenous mixture of PEG conjugated isomers (39)Additionally, due to environmental exposure and the incorporation of PEG in a variety of contemporary therapeutics, immunogenicity against PEG itself can be detrimental with the presence of anti-PEG antibodies in up to 70% of the general population (25). By modifying the amino acid sequence of the biotherapeutic directly, we can potentially avoid such limitations and reduce the complexity of the manufacturing process. Protein Re-surfacing may also represent a more economical means of bringing second line therapeutics to patients in need. By modifying the protein surface while maintaining its core function, Re-surfaced biotherapeutics may have significantly reduced rates of clinical failure in comparison to the development of an entirely new molecular entity.

Previous approaches to protein engineering such as directed evolution, random library screening and/or structure guided design have had limited capacity to search the dauntingly complex amino acid sequence space. For instance, if mutations were chosen at random, a relatively simple protein 100 amino acids in length would have 100^20^ potential sequence combinations to evaluate. Machine learning and generative biology are capable of more efficient interrogation of amino acid sequence space and simultaneous co-optimization across multiple parameters such as function, immunogenicity, and manufacturability. Efficient and effective computational design of therapeutic proteins has only recently become feasible thanks to the simultaneous advancements in computational processing power, machine learning approaches and high-throughput methods for the production and evaluation of proteins. While this study serves as a proof-of-concept for the use of Re-surfacing to alleviate the impacts of pre-existing ADAs, a separate and complementary approach would be required to reduce the immunogenicity of our engineered ASN variants. To avoid the development of new treatment emergent ADAs against the Re-surfaced proteins, one approach would be to remove or reduce the number of foreign peptides within the protein sequence that would be processed and presented on major histocompatibility complex class II (MHC-II). If MHC-II peptide presentation was avoided, you could thereby disrupt CD4+ helper T cell priming and subsequently eliminate the downstream processes required to support B cell activation, high affinity ADA formation and class switching to IgE. Engineered MHC-II epitope reduction was previously demonstrated for ASN *via* high-throughput screening of saturation mutagenesis libraries which targeted *in silico* predicted T cell epitopes by utilizing asparagine auxotrophic strain of *E. coli* (33). These efforts identified a functional ASN variant with 8 mutations which demonstrated reduced levels of treatment emergent ADAs in an HLA-DRB1*0401 transgenic mouse model. To avoid both binding by pre-existing ADA and the development of new treatment emergent ADAs, designs would need to be simultaneously co-optimized while maintaining protein function. This is a protein design challenge that machine learning approaches may be uniquely positioned to address. Our study demonstrates the capabilities of machine learning and generative biology and highlight the potential of such approaches to address previously intractable challenges in the design of novel therapeutics.

## Materials and methods

### Computational design of re-surfaced asparaginase variants

Re-surfaced ASN variants were generated using structure-based algorithms and design principles previously described by Zhou et al. and Ingraham et al. (23, 24). These approaches were utilized to introduce mutations in the reference sequence of wild-type *E. coli* asparaginase (PDB ID: 3ECA) while maintaining overall predicted structure, maximizing impact to surface exposed amino acids, and minimizing risk of disrupting protein fold or enzymatic function. The methods considered residues as designable if an amino acid had >50% relative solvent accessible surface area (RSASA) as determined by the crystal structure (freesasa.github.io), was not in direct proximity to the enzymatic active site and did not participate in monomer-to-monomer interfaces. This resulted in 85 total residues available for mutation (**Figure 1A**). The derived models were queried to produce maximally diverse sequences with a varying number of mutations constrained within the designable regions. Designed protein variants were then triaged to select 15 candidate sequences, 10 of which are reported here as the other 5 failed to express and/or showed limited to no enzymatic activity.

### Selection of re-surfaced asparaginase variants for testing

Variants were selected for large-scale production and further testing based on protein yield, activity and quality control criteria matching the specific needs of each assay. All Re-surfaced variants were required to have >90% purity by SDS-PAGE, > 90% target species by analytical size exclusion chromatography (SEC), >5nmol of protein produced from the 1mL culture (except for Resurf. ASN 7), and >50% enzymatic activity when normalized to WT E. coli ASN. Since high quality materials were often limiting, variants were also prioritized based on largest mutational distance from WT E. coli ASN. Additionally, materials utilized for in vivo studies required <100 EU/mg endotoxin and thus endotoxin removal which results in substantial loss of yield during protein purification. Resurf. ASNs 1-10 were evaluated for expression, enzymatic activity and binding to mouse and rabbit ADAs (**Figures 2**, **3**). Resurf. ASNs 1-7 were tested for binding to human ADAs (Figure 4). Resurf. ASNs 1, 2 and 6 were evaluated in the in vivo hypersensitivity mouse mode (**Figure 5**).

While prioritized for large-scale production based on high levels of mutation and low binding by mouse and rabbit ADAs, Resurf. ASNs 7 and 10 exhibited prohibitively low yields and substantial loss of materials following endotoxin removal. Resurf. ASN 10 was subsequently unavailable for human ADA binding testing and both Resurf. ASNs 7 and 10 materials were not available for in vivo testing. Resurf. ASNs 8 and 9 were closest in sequence to WT E. coli ASN, with fewer than 10% of surface exposed residues mutated. Resurf. ASNs 8 and 9 also exhibited higher binding by mouse and rabbit anti-ASN antibodies, and thus were deprioritized for large-scale production and subsequently from human sera and in vivo testing. Resurf. ASNs 1-6 were all produced at large-scale, however due to yields and the stringent endotoxin criteria only Resurf. ASNs 1, 2 and 6 were evaluated in the in vivo hypersensitivity study.

### Evaluating expression and activity of re-surfaced ASNs from bacterial lysates

Sequences for the reference and Res. ASNs were synthesized, cloned to contain a multi-histidine tag on the N-terminus for purification, and expressed in *E. coli* BL21(DE3) cells (Sigma Aldrich, St. Louis, MO) at 1mL scale to evaluate their relative expression and activity. In brief, expression vectors were transformed into *E. coli* in 96-deep well plates, cells were placed in antibiotic containing selection media and grown at 37°C overnight. The following day a starter culture was transferred to 1mL of autoinduction media (Sigma Aldrich) with antibiotic selection in 96-deep well plates and incubated while shaking overnight at 30°C. The following morning, cells were pelleted by centrifugation at 4,000g for 10 minutes and were lysed using bug buster lysis media (Sigma Aldrich). Lysed cell supernatants were harvested and subjected to expression and activity analysis. Expression was measured using a His Tag ELISA detection kit following the manufacturer’s protocols (Genscript Biotech Corporation). ASN activity was measured by first loading bacterial lysates onto anti-His tag antibody coated 96-well plates. This allowed for the capture of the recombinantly expressed ASNs while removing the WT *E. coli* ASN endogenously expressed by the host. ASN captured wells were then subjected to an ASN activity colorimetric assay following the manufacturer’s instructions (BioVision, Milpitas, CA, Cat. #K754). Selection of Re-surfaced Asparaginase Variants for Testing

Variants were selected for large-scale production and further testing based on protein yield, activity and quality control criteria matching the specific needs of each assay. All Re-surfaced variants were required to have >90% purity by SDS-PAGE, > 90% target species by analytical size exclusion chromatography (SEC), >5nmol of protein produced from the 1mL culture (except for Resurf. ASN 7), and >50% enzymatic activity when normalized to WT *E. coli* ASN. Since high quality materials were often limiting, variants were also prioritized based on largest mutational distance from WT *E. coli* ASN. Additionally, materials utilized for *in vivo* studies required <100 EU/mg endotoxin and thus endotoxin removal which results in substantial loss of yield during protein purification. Resurf. ASNs 1-10 were evaluated for expression, enzymatic activity and binding to mouse and rabbit ADAs (**Figures 2**, **3**). Resurf. ASNs 1-7 were tested for binding to human ADAs (**Figure 4**). Resurf. ASNs 1, 2 and 6 were evaluated in the *in vivo* hypersensitivity mouse mode (**Figure 5**).

While prioritized for large-scale production based on high levels of mutation and low binding by mouse and rabbit ADAs, Resurf. ASNs 7 and 10 exhibited prohibitively low yields and substantial loss of materials following endotoxin removal. Resurf. ASN 10 was subsequently unavailable for human ADA binding testing and both Resurf. ASNs 7 and 10 materials were not available for *in vivo* testing. Resurf. ASNs 8 and 9 were closest in sequence to WT *E. coli* ASN, with fewer than 10% of surface exposed residues mutated. Resurf. ASNs 8 and 9 also exhibited higher binding by mouse and rabbit anti-ASN antibodies, and thus were deprioritized for large-scale production and subsequently from human sera and *in vivo* testing. Resurf. ASNs 1-6 were all produced at large-scale, however due to yields and the stringent endotoxin criteria only Resurf. ASNs 1, 2 and 6 were evaluated in the *in vivo* hypersensitivity study.

### Large scale production and purification of re-surfaced ASNs

Proteins were produced in *E. coli* BL21(DE3) cells that were transfected with plasmid constructs as described above. The bacteria were cultivated in 1L of Terrific Broth based auto induction media after overnight expansion from glycerol stocks. After overnight induction, bacteria were harvested by centrifugation and lysed in the presence of a protease inhibitor before sonification and further centrifugation to collect protein lysates. Protein lysates were run on Cytiva HisTrap HP nickel columns (Sigma Alrich, St. Louis) where the His tagged ASNs were captured, purified, washed of possible endotoxin, and eluted, before buffer exchange into the final storage and analysis buffer of 20mM HEPES, 150mM NaCl at pH 7.4. Quality control protein analytics were performed on each preparation of materials with all test articles confirmed to exhibit ASN activity (as described below), reaching a minimum of >90% target species by analytical size exclusion chromatography, >90% purity by reduced SDS-PAGE, and <100 EU/mg endotoxin levels by Charles River endotoxin testing. If required, further removal of endotoxin was performed using high-capacity endotoxin removal spin columns (Pierce, ThermoFisher).

### Purified enzyme activity assessment

Activity of purified ASN enzymes was assessed utilizing the BioVision asparaginase activity kit. The manufacturers’ protocol for fluorometric readout was followed. In brief, samples of purified asparaginase protein were diluted to a desired concentration in assay buffer and serially titrated by two-fold dilution. The dilutions were then plated at 25µL/well in a black, opaque bottom, 96 well assay plate. The reaction mixture as described in the manufacturer’s protocol was made and added to the asparaginase dilutions immediately before placement in the Synergy Neo2 (Biotek) plate reader for kinetic readout using either the monochromator or red filter cube with excitation filter 530/25 and emission filter 590/35. Reads were made at 2-minute intervals over the course of 4 hours and data was aggregated to assess a relative enzymatic maximum velocity (relVmax) at each dilution.

### *In vivo* model studies

All animal protocols were reviewed and approved by the Institutional Animal Care and Use Committee of Charles River Labs (Worcester, MA) in accordance with the Association for the Assessment and Accreditation of Laboratory Animal Care Guidelines.

### Generation of mouse anti-asparaginase plasma

To generate a source of anti-asparaginase mouse plasma, groups of female Balb/c mice between the ages of 7 to 9 weeks were immunized with WT *E. coli* ASN. Aluminum hydroxide and magnesium hydroxide (Imject^R^ Alum, ThermoFisher) adjuvant was mixed 1:1 with 15µg WT ASN suspended in 100µl of 0.1 M phosphate-buffered saline (PBS), pH 7.2 (ThermoFisher). The 200µl of ASN and Alum mix was administered intraperitoneally three times every seven days over the course of three weeks. Plasma was harvested following terminal cardiac bleeds 3 days post the final dose. Plasma was then pooled, aliquoted to prevent repeat freeze/thaws and stored at -80°C.

### Primary patient specimens

All patients provided written informed consent in accordance with the Declaration of Helsinki, and studies were reviewed and approved by the Institutional Review Board at the Dana-Farber Cancer Institute (DFCI, Boston, MA).

### Selection of plasma and serum

Banked plasma and serum samples from adult patients with ALL were identified from an institutional tissue bank. Patients who had hypersensitivity reactions in the context of ASN exposure were identified through electronic health record abstraction. Hypersensitivity reactions were graded for severity according to the following features: clinical anaphylaxis, skin/mucosal involvement (hives/swelling/injection site erythema), airway compromise (dyspnea/bronchospasm/difficulty swallowing), or gastrointestinal symptoms and signs (abdominal pain/diarrhea). We retrieved twenty such samples obtained from patients following a documented severe hypersensitivity reaction in the context of ASN treatment (median interval, 115 days). Patient samples were then tested for high antibody titers against WT *E. coli* ASN (see *Anti-asparaginase antibody binding measurements* below). Eleven samples containing high anti-ASN titers were then selected for further evaluation. One of the eleven patient samples exhibited the highest levels of binding across all Resurf. ASN variants, with several variants showing a 3 to 4-fold increase in binding for that donor which fell far outside the expected distributions. Following outlier analyzes using both Grubbs and ROUT methodologies with a false-discovery rate of 0.05 using GraphPad Prism (Dotmatics) this sample was detected as a significant outlier for 3 to 4 of the Resurf. ASN variants, respectively. Due to the irregularity of this data and potential unknown biological confounders inherent to patient derived materials, this sample was excluded from analyzes.

### Anti-asparaginase antibody binding measurements

An enzyme-linked immunosorbent assay (ELISA) was performed to quantify the binding of antibodies from murine, rabbit, and human patient biospecimens against WT *E. coli* ASN, *Erwinia* ASN and Re. ASN variants. Ninety-six-well plates were coated with 50µL of 5µg/mL *E. coli*, *Erwinia*, or Res. ASN diluted in ELISA Coating Buffer (Biolegend) and incubated overnight at 4°C. Wells were decanted and blocked for 90 minutes with 250µL of ELISA Assay Diluent (Biolegend) and PBS, then washed three times with PBS Tween-20 (ThermoFisher). Samples containing murine, rabbit or human ADAs were added and incubated for 60 minutes at 37°C. Plates were decanted and washed. Anti-IgG secondary reagents utilizing HRP were added to each well and incubated for 60 minutes at 37°C. Plates were decanted and washed. Finally, 50µL of 1-step Ultra TMB (ThermoFisher) was added to each well, incubated for 5 minutes at room temperature and then 50µL of Stop Solution (ThermoFisher) was added to each well and the absorbance measured immediately after at 450nm on the Envision Multimode Plate Reader (PerkinElmer). All experimental and control wells were run in technical triplicate. Each plate contained control wells incubated with Secondary antibody alone to establish background signal level. The average background was then subtracted from signals obtained from all experimental wells on the associated plate.

To detect binding by murine ADAs, polyclonal goat anti-Mouse IgG-HRP (Abcam) was diluted 1:10,000 in ELISA Assay Diluent (Biolegend), with 50µL added to each well. To detect rabbit ADAs, polyclonal goat anti-rabbit H+L IgG-HRP (Abcam) was diluted 1:1,000 in ELISA Assay Diluent buffer, with 50µL added to each well. To detect binding by human ADAs, polyclonal goat anti-Human H+L chain IgG-HRP (Promega) diluted 1:10,000 in ELISA Assay Diluent, with 50µL added to each well.

Assessment of ADA titer was performed by serially diluting murine or human samples in ELISA Assay Diluent buffer. Titer calculation entailed taking the highest dilution (lowest concentration of sample) in which background normalized signal for an experimental well was at least 3-fold greater than healthy pooled normal murine plasma, human serum, or human plasma, as appropriate.

### HIS quantification of ASN coating efficiency by ELISA

An anti-histidine ELISA was run to assess and control for the relative quantity of HIS-tagged protein present on the anti-ASN binding ELISA plates described above. The anti-HIS ELISA followed the same protocol as described above. Mouse anti-HIS IgG1 antibody (BioXcell, clone 6-His) was used at 4µg/mL for the primary incubation and 1:10,000 polyclonal goat anti-Mouse IgG-HRP (Abcam) for the secondary incubation. The relative absorbances of Res. ASNs to *E. coli* ASN were applied as normalization factors to the absorbance measurements in the anti-ASN IgG ELISA.

### Murine *in vivo* study assessing ASN induced hypersensitivity

To evaluate the biological impact of protein Re-surfacing, a murine hypersensitivity model was utilized. Weight monitored female Balb/c mice between the ages of 7 and 9 weeks were sensitized with WT ASN before subsequent challenge with WT ASN, *Erwinia* ASN, vehicle alone, or 3 Res. ASN variants. All mice were bled prior to the start of treatment and plasma was isolated to assess baseline plasma binding to the test articles. After the initial bleed mice were sensitized with WT ASN *via* intravenous injections of 15µg of protein formulated in PBS in a 200µL volume on days 0, 7 and 14. Throughout the experiment, hypersensitivity response was scored on a 5-point scale *via* clinical observation for the 30 minutes proceeding each dosing (27). Mice were given a score of 0 if there were no signs of shock, 1 for mild shock including ruffled fur, itching, dyspnea and decreased spontaneous movement, 2 for moderate shock including prostration, sluggish gait, and slight activity after prodding, 3 for severe shock including paresis, no activity following prodding with or without convulsions, or 4 for mortality within 30 minutes. Technicians carrying out the study and measuring hypersensitivity responses were blinded to the nature of the materials being administered to minimize observational bias. Following the sensitization phase, experimental subgroups were stratified to ensure equal distribution and mean of hypersensitivity score per subgroup. On Day 20, prior to the initiation of the challenge phase, mice were bled and plasma was isolated. During the challenge phase, mice received three doses of vehicle alone, WT ASN, *Erwinia* ASN, or one of three Res. ASNs. Each dose occurred seven days apart on days 21, 28, and 35, and contained 15µg of test protein in a 200µL volume. At the completion of the study, 3 days post the final challenge dose, mice were euthanized and terminally bled *via* cardiac puncture. Plasma samples from the mice were assessed for ADA response *via* mouse IgG ELISA as described above.

## Data availability statement

The original contributions presented in the study are included in the article/**Supplementary Material**. Further inquiries can be directed to the corresponding authors.

## Ethics statement

The studies involving human participants were reviewed and approved by Institutional Review Board at the Dana-Farber Cancer Institute (DFCI, Boston, MA). The patients/participants provided their written informed consent to participate in this study. The animal study was reviewed and approved by Institutional Animal Care and Use Committee of Charles River Labs (Worcester, MA). Written informed consent was obtained from the individual(s) for the publication of any potentially identifiable images or data included in this article.

## Author contributions

Conceptualization: MF, GG, and DL; computational design: MF and GG; investigations: AB, HA, RB, MH, and DL; protein expression and purifications: AB, BM, LX, SP, JG, and RB; data analysis: AB, HA, and DL; writing (review and editing): AB, HA, MM, KH, and DL; writing (original draft): AB, HA, MM, and DL; supervision: JG, GG, KH, and DL. All authors contributed to the article and approved the submitted version.

## Acknowledgments

We thank Alison Tisdale, Joe Cabral and Jordi Mata-Fink for their contributions to the initiation of this project and establishment of the collaboration with the Dana Farber Cancer Institute. We thank Kenneth Manning for his contributions towards the expression and purification of several proteins utilized in this study.

## Conflict of interest

AB, MF, BM, LX, SP, JG, RB, MH, GG, KH, and DL have or had stock options or ownership in Generate Biomedicines. MF, GG, and DL are inventors on a patent application related to this work filed by Generate Biomedicines PCT/US2021/071931, filed on 19th of October of 2021, which is pending and unpublished.

The remaining authors declare that the research was conducted in the absence of any commercial or financial relationships that could be construed as a potential conflict of interest.

## Publisher’s note

All claims expressed in this article are solely those of the authors and do not necessarily represent those of their affiliated organizations, or those of the publisher, the editors and the reviewers. Any product that may be evaluated in this article, or claim that may be made by its manufacturer, is not guaranteed or endorsed by the publisher.
