# Supplementary material for: Protein re-surfacing of E. coli L-Asparaginase to evade pre-existing anti-drug antibodies and hypersensitivity responses
Source: Front Immunol. 2022 Dec 7;13:1016179. doi: 10.3389/fimmu.2022.1016179 (PMC9767956; doi:10.3389/fimmu.2022.1016179)
Supplement: Supplementary file 1 [file DataSheet_1.pdf]

Supplementary Material

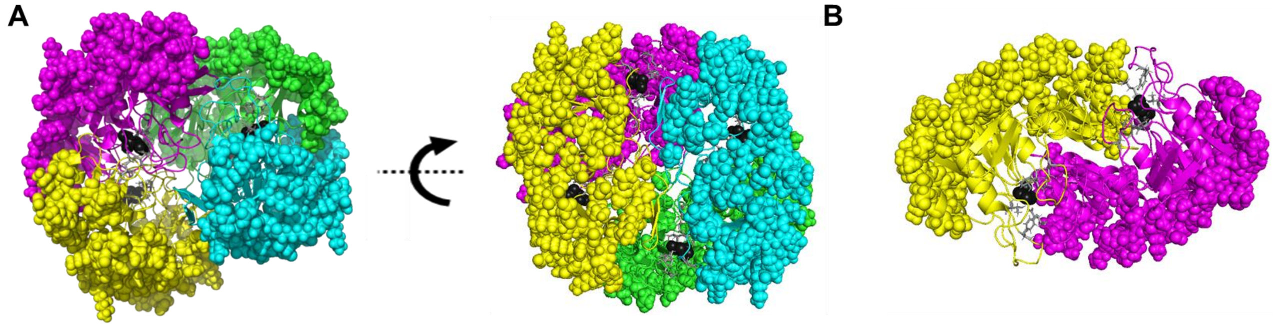

SUPPLEMENTARY FIGURE 1. Computational design space for creating Re-surfaced ASN variants. Variant proteins were designed referencing the wild-type *E. coli* asparaginase (WT ASN) heterotetramer crystal structure (PDB ID: 3ECA). Amino acid positions were targeted for mutation if they were >50% solvent accessible, in direct proximity to a solvent accessible residue and not in direct proximity to either the enzymatic core or monomer-monomer interfaces. Each of the four monomers making up the heterotetramer are shown in yellow, magenta, aquamarine or green. Surface amino acids open to mutation are denoted by spheres. Within the interior, the active site is denoted by black spheres (substrate) and grey sticks (active site residue side chains). (**A**) Complete heterotetramer is shown. (**B**) Two monomers making up two complete active sites is shown.

ID	Sex	Age	Diagnosis	Cytogenetics	Therapy Regimen(s) prior to Reaction	Lines of Therapy before time of sample	ASN Formulation	Days between ASN Tx sta and Allergic Reaction
Plasma #1	F	49	B-ALL	46,XX,t(4;11)(q21;q23)[12]/46,XX[8]	DFCI Study 15-709	1	PEG	107
Plasma #2	M	49	Pre B-ALL	47,XY,t(2;14)(p13;q32),+22[12]/46,XY[8]	Larsen Regimen	2	PEG	51
Plasma #3	M	31	Early T-cell Precursor ALL.	CALM/AF10 translocation t(10;11), del5q abnormality	Larsen Regimen	1	E. coli	15
Plasma #4	F	51	Ph- B ALL	46,XX[20]	Larsen Regimen	1	PEG	14
Plasma #5	M	58	Ph- Pre-B ALL	46,XX[20]	Larsen Regimen	1	PEG	14
Serum #1	M	31	Pre-T ALL	46,XX[20]	CAPP (thymoma misdiagnosis) > Larsen Regimen	2	E. coli	0
Serum #2	M	57	Ph- Pre-B ALL	35-37,XY,-4,-7,-14,-15,-17[cp4]/46,XY[10].nuc ish 4cen(CEP4x1), 7cen (D7Z1x1)	Larsen Regimen	1	E. coli	32
Serum #3	M	55	Ph- Pre-B ALL	45,XY,-7[4].nuc ish 7cen(D7Z1x1)	Larsen Regimen	1	E. coli	41
Serum #4	F	42	Ph- PAS+ Pre-B ALL, FAB-L2 type	35,XX,-3,-4,-5,-7,-9,-13,-14,-15,-16,-17,-20[cp6]	Larsen Regimen	1	E. coli	36
Serum #5	M	19	Ph- Pre-B ALL	46,XY,del(9)(p21)[11]/46,idem,?del(6)(q?23q?24)[2]/46,XY[7].nuc ish 9q34(ABLx2),22q11.2(BCRx2)	POG 9900	1	PEG	0
Serum #6	M	48	Ph- Pre-B ALL	46,XY.nuc ish 9q34(IGHx3-4)[70/100]	Larsen Regimen	1	E. coli	32

SUPPLEMENTARY TABLE 1. Detailed summary of patient characteristics for samples containing anti-ASN ADAs. Data was found through clinical records on EPIC. Best estimates are given for cases with unspecified asparaginase treatment or reaction dates.

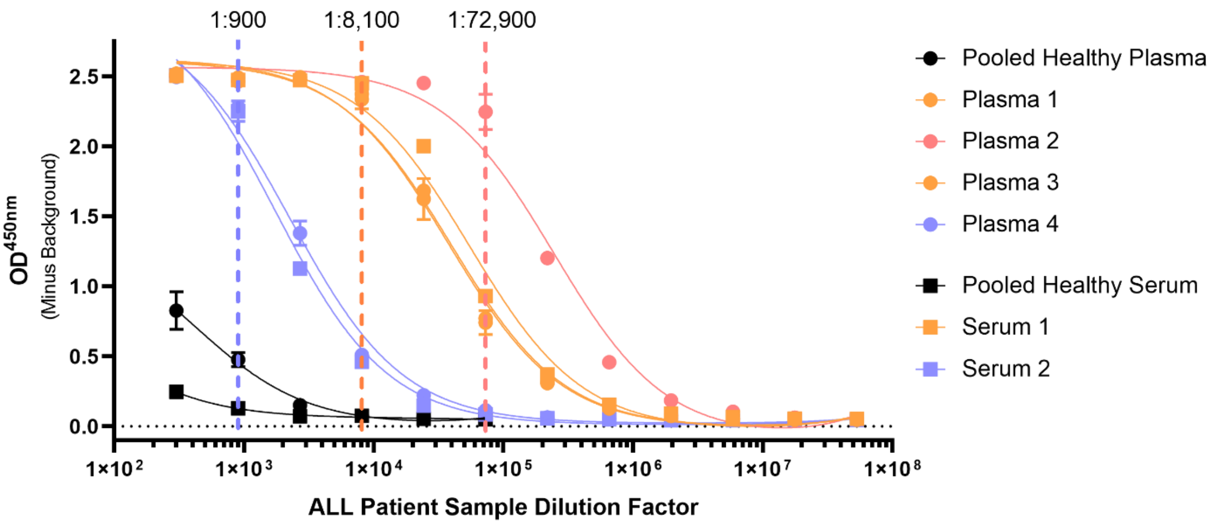

SUPPLEMENTARY FIGURE 2. Identification of optimal human sample concentration for use in measuring relative binding of human ADAs to ASNs. Raw OD^{450} signal from representative ADA titering ELISAs. Samples were serially diluted and tested for binding against WT ASN. Pooled healthy volunteer plasma or serum (black circle and square, respectively) and a representative subset of plasma and serum samples from patients exhibiting high titers of anti-ASN ADAs is shown. Dotted lines indicate specific dilutions that were used for the indicated patient samples in subsequent measures of relative binding against Resurf. ASN variants.

	Plasma Sample Donors					Serum Sample Donors				
**Protein**	**1**	**2**	**3**	**4**	**5**	**6**	**7**	**8**	**9**	**10**
**WT *E. coli* ASN**	100.0	100.0	100.0	100.0	100.0	100.0	100.0	100.0	100.0	100.0
***Erwinia* ASN**	2.3	0.4	0.0	0.0	0.0	0.1	11.2	0.4	7.0	0.0
**Resurf. ASN 7**	18.2	5.5	0.0	0.0	0.0	2.0	17.0	53.3	7.2	0.0
**Resurf. ASN 3**	65.2	35.1	76.4	0.6	76.3	107.6	63.4	75.6	31.1	84.3
**Resurf. ASN 5**	44.7	39.9	72.0	21.9	106.5	17.9	27.4	70.8	24.0	64.7
**Resurf. ASN 1**	64.4	51.5	84.0	15.6	120.9	98.0	50.4	94.2	45.5	88.8
**Resurf. ASN 2**	81.8	37.2	67.4	52.0	55.3	67.7	47.8	84.5	33.8	80.9
**Resurf. ASN 4**	63.0	43.9	49.4	21.3	62.9	95.9	48.9	83.5	23.7	74.0
**Resurf. ASN 6**	89.3	25.3	1.9	10.5	0.0	130.1	118.5	106.6	69.2	10.8

Supplementary Table 2. Average patient plasma and serum sample anti-asparaginase IgG antibody binding levels relative to *E. coli* asparaginase. ELISA absorbance measurements were conducted in triplicate. Values shown are averaged and normalized to the binding observed for WT *E. coli* ASN wells on a per donor basis. See Figure 4.

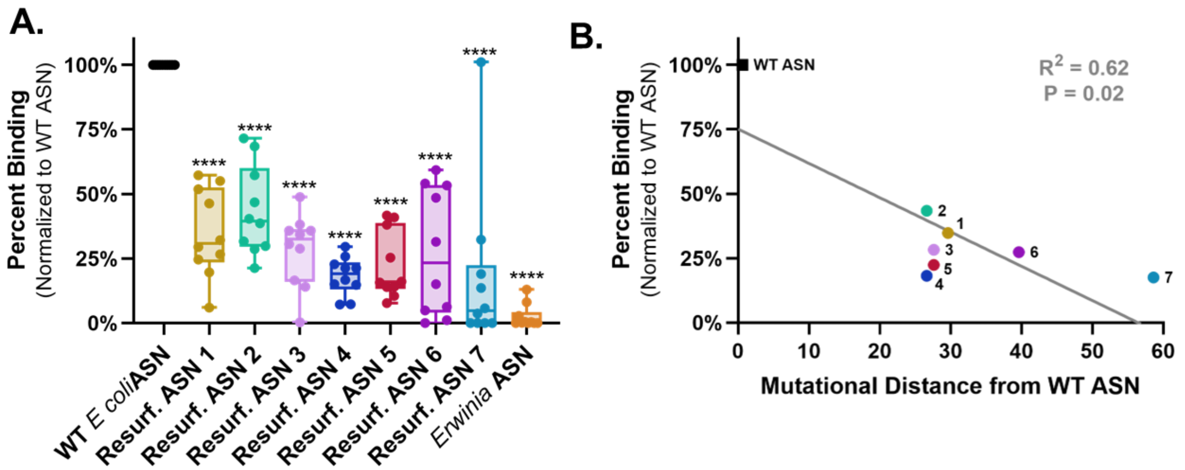

SUPPLEMENTARY FIGURE 3. Relative human ADA binding to Re-surfaced ASNs normalized to His-tag quantification. Anti-ASN ADA titers were quantified against WT ASN for each sample via ELISA with the dilution exhibiting 80% of max binding signal utilized to measure relative binding to Resurf. ASN variants (See Supplemental Figure 1). Relative abundance of each ASN control or variant was quantified via an anti-His ELISA since each recombinant protein contained a Histidine tag. (**A**) Relative binding of human serum or plasma samples against Resurf. ASNs or *Erwinia* ASN control was assessed by IgG ELISA. Data was subsequently normalized to the relative abundance calculated via anti-His ELISA. Each dot is the mean of an individual patient sample tested in technical triplicate. All ASN variants exhibited significant reductions in normalized binding via one-way analysis of variance (ANOVA), ****P<0.0001. (**B**) The mean normalized percent binding across all patient samples to each ASN is plotted against the mutational distance of the molecule to WT ASN and shows a significant negative correlation via linear regression analysis, R^2 = 0.62, P = 0.02. Data are representative of N = 3 independent experiments.

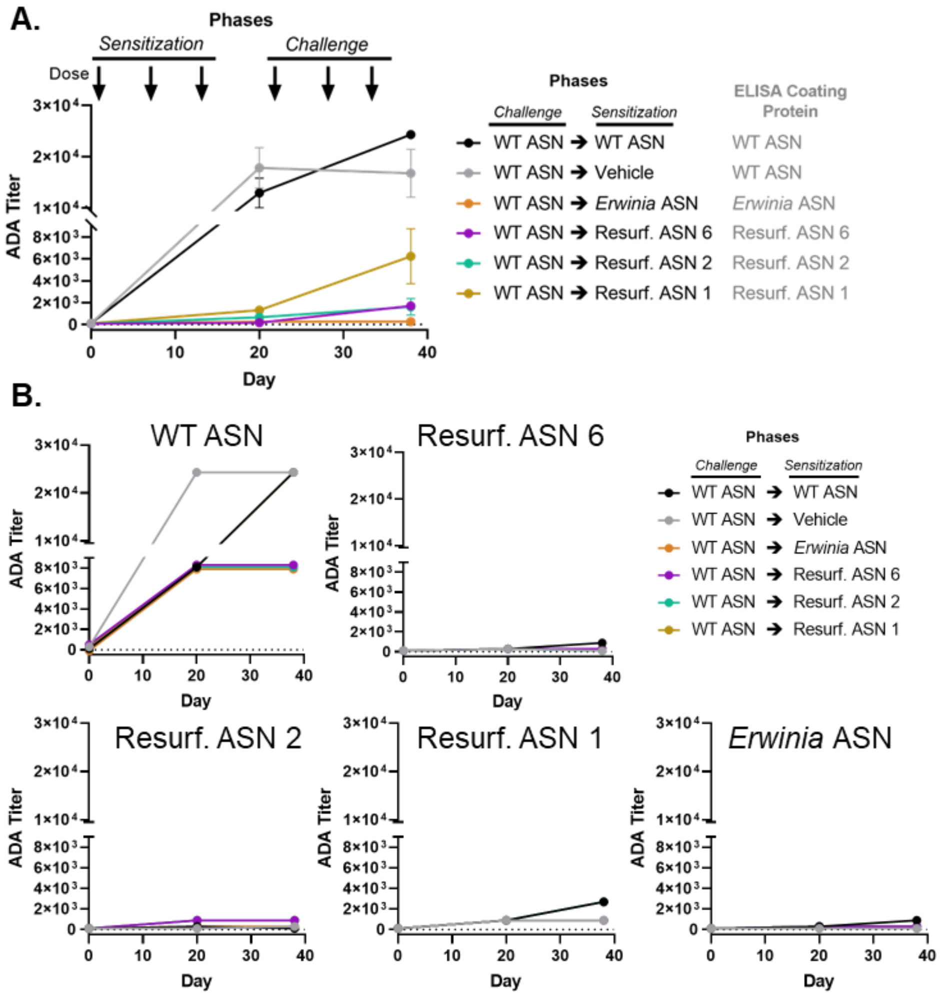

SUPPLEMENTARY FIGURE 4. Anti-ASN ADA titers from hypersensitivity *In vivo* study. Balbc mice from the hypersensitivity *in vivo* study were bled prior to onset of sensitization phase on day 0, after sensitization on Day 20, and finally after challenge phase at the completion of the study on day 38. Plasma was isolated from blood draws and frozen for later analysis. An ELISA was conducted to assess ADA titers against each experimental group's respective challenge phase treatment molecule. Binding measurements were performed in technical triplicate with positive titer being determined as the highest dilution (lowest sample concentration) in which ELISA signal was higher than healthy pooled normal mouse serum. (**A**) The mean ADA titer from individual mice at the indicated time point for each experimental group is shown. Groups are colored according to the challenge phase dose protein and subsequent target protein evaluated for binding by ELISA. (**B**) The ADA titers measured from pooled serum from the indicated experimental group and assayed for binding against the indicated challenge protein, irrespective of whether that experimental group was dosed with said protein. This analysis enabled assessment of ADA epitope spread across experimental groups and potential impact of anti-WT ASN ADA titer following shift to treatment with Resurf. or *Erwinia* ASN. The color of the data points and lines indicated the experimental group and the target protein listed above each graph indicates the protein for which ADA binding is being measured.

```
WT ASN        LPNITILATGGTIAGGGDSATKSNYTVGKVGVENLVNAVPQLKDIANVKGEQVVNIGSQD   60
Res. ASN 8    LPNITILATGGTIAGGGDSATKSNYTVGKVGVENLVNAVPQLKDIANVKGEQVKNIGSQD   60
Res. ASN 3    LKNITILATGGTIAGGGDSATKSNYTVGKVGVENLVKAVPQLADIANVKGEQVKNIGSQD   60
Res. ASN 5    LKNITILATGGTIAGGGDSATKSNYTVGKVGVDKLVEAVPQLKDIANVKGEQVKNIGSQD   60
Res. ASN 1    IKKITILATGGTIAGGGDSATKSNYTVGKVGVEELVERVPQLKDIAEVKGEQVMNIGSQD   60
Res. ASN 10   IKNITILATGGTIAGGGDSATKSNYTVGKVGVENLVKAVPQLKDIANVRGEQVKNIGSQD   60
Res. ASN 2    LPNITILATGGTIAGGGDSATKSNYTVGKVGVDNLVALVPQLKDIANVKGEQVKNIGSQD   60
Res. ASN 9    LPNITILATGGTIAGGGDSATKSNYTVGKVGVENLVKAVPQLKDIANVKGEQVKNIGSQD   60
Res. ASN 7    KKKITILATGGTIAGGGDSATKSNYTVGKVGVDELIARVPQLADIAEVDGEQVMNIGSQD   60
Res. ASN 4    LPNITILATGGTIAGGGDSATKSNYTVGKVGVENLVAAVPQLKDIANVKVEQVKNIGSQD   60
Res. ASN 6    MKKITILATGGTIAGGGDSATKSNYTVGKVGVDELVAGVPQLADIADVTAEEVRNIGSQD   60
                :*****************************::*:  **** ***:*  *:* ******

WT ASN        MNDNVWLTLAKKINTDCDKTDGFVITHGTDTMEETAYFLDLTVKCDKPVVMVGAMRPSTS   120
Res. ASN 8    MNDNVWLTLAKKINTDCDKTDGFVITHGTDTMEETAYFLDLTVKCDKPVVMVGAMRPSTS   120
Res. ASN 3    MNDEVWLKLAKKINDDCDKTDGFVITHGTDTMEETAYFLDLTVKCDKPVVMVGAMRPSTS   120
Res. ASN 5    MNDEVWLKLALKINTDCDKTDGFVITHGTDTMEETAYFLDLTVKCDKPVVMVGAMRPSTS   120
Res. ASN 1    MNDEVWLKLAKKINEDCDKTDGFVITHGTDTMEETAYFLDLTVKCDKPVVMVGAMRPSTS   120
Res. ASN 10   MNDEVWLKLAEKINKDCDKTDGFVITHGTDTMEETAYFLDLTVKCDKPVVMVGAMRPSTS   120
Res. ASN 2    MNDEVWLKLAKKINDDCDKTDGFVITHGTDTMEETAYFLDLTVKCDKPVVMVGAMRPSTS   120
Res. ASN 9    MNDNVWLRLAKKINDDCDKTDGFVITHGTDTMEETAYFLDLTVKCDKPVVMVGAMRPSTS   120
Res. ASN 7    MNDEVWLKLAKRINELCEQYDGFVITHGTDTMEETAYFLDLTVKCSKPVVMVGAMRPSTS   120
Res. ASN 4    MNDEVWLKLAKKINDDCDKTDGFVITHGTDTMEETAYFLDLTVKCDKPVVMVGAMRPSTS   120
Res. ASN 6    MNDEVWLELARKINEDCEKTDGFVITHGTDTMEETAYFLDLTVTCNKPVVMVGAMRPSTS   120
              ***:*** ** :**  *:: ***********************.*.**************
```

SUPPLEMENTARY FIGURE 4. Caption below

```
WT ASN          MSADGPFNLYNAVVTAADKASANRGVLVVMNDTVLDGRDVTKTNTTDVATFKSVNYGPLG   180
Res. ASN 8      MSADGPFNLYNAVVTAADKASANRGVLVVMNDTVLDGRDVTKTNTTDVATFKSVNYGPLG   180
Res. ASN 3      MSADGPFNLYNAVVTAADQRSAGRGVLVVMNDTVLDGRDVTKTNTTDVATFKSVNYGPLG   180
Res. ASN 5      MSADGPFNLYNAVVTAADPASAGRGVLVVMNDTVLDGRDVTKTNTTDVATFKSVNYGPLG   180
Res. ASN 1      MSADGPFNLYNAVVTAADQRSAGRGVLVVMNDTVLDGRDVTKTNTTDVATFKSVNYGPLG   180
Res. ASN 10     MSADGPFNLYNAVVTAADQRSAGRGVLVVMNDTVLDGRDVTKTNTTDVATFKSVNYGPLG   180
Res. ASN 2      MSADGPFNLYNAVVTAADERSAGRGVLVVMNDTVLDGRDVTKTNTTDVATFKSVNYGPLG   180
Res. ASN 9      MSADGPFNLYNAVVTAADKRSAGRGVLVVMNDTVLDGRDVTKTNTTDVATFKSVNYGPLG   180
Res. ASN 7      MSADGPFNLYNAVVTAAHPESKGQGVLVVMNDTVLDGRDVTKTNTTDVATFKSVNYGPLG   180
Res. ASN 4      MSADGPFNLYNAVVTAADKRSANRGVLVVMNDTVLDGRDVTKTNTTDVATFKSVNYGPLG   180
Res. ASN 6      MSADGPFNLYNAVVTAADDASAGRGVLVVMNDTVLDGRDVTKTNTTDVATFKSVNYGPLG   180
                *****************.  * .:************************************

WT ASN          YIHNGKIDYQRTPARKHTSDTPFDVSKLNELPKVGIVYNYANASDLPAKALVDAGYDGIV   240
Res. ASN 8      YIHNGKIDYQRTPARKHTSDSEFDVSKLNELPKVGIVYNYANASDLPAKALIDAGYDGIV   240
Res. ASN 3      YIHNGKIDYQRTPARPHTPDTEFDVSKLTSLPKVGIVYNYANASVLPAKALVDAGYDGIV   240
Res. ASN 5      YIHNGKIDYQRTPARPHTLATEFDVSSLTELPKVGIVYNYANASVLPAKALVDAGYDGIV   240
Res. ASN 1      YIHNGKIDYQRTPARPHTPDTEFDVSKLTELPKVGIVYNYANASSLPAKALVEAGYDGIV   240
Res. ASN 10     YIHNGKIDYQRTPARLHTPLTEFDVSSLDALPKVGIVYNYANASALPAKALVDAGYDGIV   240
Res. ASN 2      YIHNGKIDYQRTPARKHTPLTEFDVSNLTELPKVGIVYNYANASDLPAKALVDAGYDGIV   240
Res. ASN 9      YIHNGKIDYQRTPARKHTPLTPFDVSKLNELPKVGIVYNYANASSLPAKALVDAGYDGIV   240
Res. ASN 7      YIHNGKIDYQRQPARPHTLASEFDVSKIEELPKVGIVYNYANAFVLPAEALIKAGYDGIV   240
Res. ASN 4      YIHNGKIDYQRTPARLHTLETEFDVSKLDELPKVGIVYNYANASVLPAKALVDAGYDGIV   240
Res. ASN 6      YIHNGKIDYQRTPARKHTKDTPFDVSGLTSLPKVGIVYNYANASSLPAKALVEAGYDGIV   240
                *********** *** **  : **** :  *************  ***:**:.*******
```

SUPPLEMENTARY FIGURE 4. Caption below

```
                                         $
                                    _____
                                      #
                           ___________
WT ASN          SAGVGNGNLYKSVFDTLATAAKTGTAVVRSSRVPTGATTQDAEVDDAKYGFVASGTLNPQ   300
Res. ASN 8      SAGVGNGNLYKSVFDTLATAAKDGTAVVRSSRVPTGATTQDAEVDDAKYGFVASGTLNPQ   300
Res. ASN 3      SAGVGNGNLYKEVFKTLAEAAKNGTAVVRSSRVPTGATTQDAEVDDAKYGFVASGTLNPQ   300
Res. ASN 5      SAGVGNGNLYKEVFDTLAKAAEDGTAVVRSSRVPTGATTQDAEVDDAKYGFVASGTLNPQ   300
Res. ASN 1      SAGVGNGNLYKSVFDTLAKAAEDGTAVVRSSRVPTGATTQDAEVDDAKYGFVASGTLNPQ   300
Res. ASN 10     SAGVGNGNLYKEVFQTLAQAAADGTAVVRSSRVPTGATTQDAEVDDAKYGFVASGTLNPQ   300
Res. ASN 2      SAGVGNGNLYKLVFITLATAAKNGTAVVRSSRVPTGATTQDAEVDDEKYGFVASGTLNPQ   300
Res. ASN 9      SAGVGNGNLYKSVFDTLATAAKNGTAVVRSSRVPTGATTQDAEVDDAKYGFVASGTLNPQ   300
Res. ASN 7      SAGVGNGNLYKEVFKALKEAAKNGIAVVRSSRVPTGATTQDAEVDDEQYGFVASGTLNPQ   300
Res. ASN 4      SAGVGNGNLYKEVFKTLAEAARRGTAVVRSSRVPTGATTQDAEVDDEKYGFVASGTLNPQ   300
Res. ASN 6      SAGVGNGNLYKEVFETLAEAAKNGTAVVRSSRVPTGATTQDAEVDDAAYGFVASGTLNPQ   300
                *********** ** :*  **  * *********************  ************

WT ASN          KARVLLQLALTQTKDPQQIQQIFNQY 326
Res. ASN 8      KARVLLQLALIQTKDPQQIQQIFNQY 326
Res. ASN 3      KARVLLQLALTKTNDPEEIQRIFDTY 326
Res. ASN 5      KARVLLQLALTKTKDPEEIQKIFDTY 326
Res. ASN 1      KARVLLQLALTKTKDPEEIQRIFDTY 326
Res. ASN 10     KARVLLQLALTKTKDPEEIQEIFDRY 326
Res. ASN 2      KARVLLQLALTKTNDPREIQEIFDTY 326
Res. ASN 9      KARVLLQLALTKTNDPQQIQQIFNQY 326
Res. ASN 7      KARVLLQLALVKTNDRREIQRIFDTY 326
Res. ASN 4      KARVLLQLALTKTNDPREIQEIFRRY 326
Res. ASN 6      KARVLLQLALTLTKDPAAIQAIFDEY 326
                **********  *:*   ** **  *
```

SUPPLEMENTARY FIGURE 5. Resurfaced ASN modifications for previously identified B cell epitopes on *E. coli* ASN. A multiple sequence alignment is shown for each of the 326 amino acids (minus the cleaved secretion signal sequence and histidine tags) for WT *E. coli* ASN (top line in bold) and Resurf. ASN variants evaluated in this study. Previously reported human ADA epitopes noted above sequences with red lines indicating residues within the peptide that have been modified by one or several Resurf. ASNs. Citations ([#] = Werner, *Biol. Chem.* 2005; [$] = Jianhua, *Mol Biotechnol.* 2006). Clusta omega consensus symbols shown on bottom ("*" = fully conserved residue, ":" = conservation between groups of strongly similar properties, "." = conservation between groups of weakly similar properties). Bold red amino acids indicate a position mutated away from WT ASN sequence for the indicated Resurf. ASN.
